# The response of total testing process in clinical laboratory medicine to COVID-19 pandemic

**DOI:** 10.11613/BM.2021.020713

**Published:** 2021-06-15

**Authors:** Funda Eren, Merve Ergin Tuncay, Esra Firat Oguz, Salim Neselioglu, Ozcan Erel

**Affiliations:** 1 Central Biochemistry Laboratory, Ankara City Hospital, Ankara, Turkey; 2 Department of Biochemistry, Ankara Yıldırım Beyazıt University Faculty of Medicine, Ankara, Turkey

**Keywords:** COVID-19, quality indicator, pandemic, total testing process

## Abstract

**Introduction:**

Following a pandemic, laboratory medicine is vulnerable to laboratory errors due to the stressful and high workloads. We aimed to examine how laboratory errors may arise from factors, *e.g.,* flexible working order, staff displacement, changes in the number of tests, and samples will reflect on the total test process (TTP) during the pandemic period.

**Materials and methods:**

In 12 months, 6 months before and during the pandemic, laboratory errors were assessed via quality indicators (QIs) related to TTP phases. QIs were grouped as pre-, intra- and postanalytical. The results of QIs were expressed in defect percentages and sigma, evaluated with 3 levels of performance quality: 25^th^, 50^th^ and 75^th^ percentile values.

**Results:**

When the pre- and during pandemic periods were compared, the sigma value of the samples not received was significantly lower in pre-pandemic group than during pandemic group (4.7σ *vs.* 5.4σ, P = 0.003). The sigma values of samples transported inappropriately and haemolysed samples were significantly higher in pre-pandemic period than during pandemic (5.0σ *vs.* 4.9σ, 4.3σ *vs.* 4.1σ; P = 0.046 and P = 0.044, respectively). Sigma value of tests with inappropriate IQC performances was lower during pandemic compared to the pre-pandemic period (3.3σ *vs.* 3.2σ, P = 0.081). Sigma value of the reports delivered outside the specified time was higher during pandemic than pre-pandemic period (3.0σ *vs.* 3.1σ, P = 0.030).

**Conclusion:**

In all TTP phases, some quality indicators improved while others regressed during the pandemic period. It was observed that preanalytical phase was affected more by the pandemic.

## Introduction

Cases of COVID-19 were reported in more than one hundred countries throughout the world and has resulted in a pandemic (*1*, *2*). As the whole world continues to fight the coronavirus, the epidemic poses a challenge for individuals, communities, as well as health systems, healthcare professionals, and laboratories (*3*). Clinical laboratory owns critical roles in managing COVID-19. Laboratory tests are used for diagnosing, prognosticating, and therapeutic monitoring of COVID-19 (*4*-*9*).

Although the importance of laboratory medicine in the healthcare system was emphasized with the pandemic, laboratory testing is a quite complex process (*4*). The total testing process (TTP) involves several steps, each of which can cause errors. Errors in laboratory testing have a substantial influence on patient outcomes (*10*, *11*). In laboratory practice, the TTP is classified into three essential phases: pre-analytical, analytical, and postanalytical steps (*12*). Quality indicators (QIs) are recognized as cornerstone tools for the quality of laboratory systems that can be measured to evaluate each step of TTP. The use of QIs in laboratory medicine enables to identify of error rates and reduce or prevent error risks regarding to patient safety (*13*, *14*).

Natural disasters involving earthquakes, tsunamis, fire, or epidemics usually cause health, and safety difficulties (*15*). During the pandemic period laboratory errors may occur due to various factors such as flexible working order applied in the laboratory with the pandemic, staff shifting to different departments, insufficient number of staff due to COVID-19 infection, change of device users, stressful and increased workload, need for additional devices in view of an increased number of requests in some test groups, and increased panic values derive from patients with COVID-19 were considered. When faced with environmental factors such as pandemics and natural disasters, there is almost no study done on what kind of problems can arise in clinical laboratories (*16*, *17*). This study aimed to examine how laboratory errors that may arise from factors such as flexible working order, staff displacement, changes in the number of tests, and samples will reflect on the TTP during the pandemic period.

## Material and Methods

### Study design

The study was conducted in Turkey’s largest hospital pandemic. In order to examine the effect of the pandemic on the total test process, a total of 12 months, 6 months before the pandemic, and 6 months after the pandemic onset, were evaluated using QIs. COVID-19 was declared a pandemic by the World Health Organization (WHO) on March 11, 2020, when the first case was seen in our country. For this reason, in our study, the month of March 2020 was accepted as the beginning of the pandemic, and laboratory errors in the TTP of 6 months before March 2020 and 6 months after March 2020 were analysed. Data were collected retrospectively from the electronic laboratory information management system. In our hospital, laboratory errors in TTP are regularly recorded. Parameters in preanalytical, analytical, and postanalytical processes are evaluated monthly by laboratory experts. In addition, regulatory preventive actions are organized when necessary. All these data can be accessed from the electronic laboratory information management system. Data on quality indicators are transmitted monthly to the quality unit of our hospital by laboratory quality officers. The hospital quality unit periodically conducts in-hospital quality control. Moreover, productivity and quality control to all hospitals and laboratories in Turkey is carried out twice a year by the health ministry.

Quality indicators were chosen from a common model of QIs set by the International Federation of Clinical Chemistry and Laboratory Medicine (IFCC) (*13*, *18*, *19*). QIs were categorized in accordance with the main TTP phases as shown in Table 1. Data on inappropriate testing requests were collected in close collaboration with clinicians. Samples with free haemoglobin (Hb) were detected by automated haemolytic index (PreHemI). The inappropriateness of sample transportation refers to samples that are damaged during transportation (pre-DamS). The study procedure was set in accordance with the basis of the Helsinki Declaration and confirmed by the local ethics board.

**Table 1:** Quality indicators selected for the study

**Preanalytical phase**
Misidentification errors:
Misidentified requests (Pre-MisR)
Misidentified samples (Pre-MisS)
Inappropriate test requests (Pre-OffDE)
Incorrect sample type:
Wrong or inappropriate type of samples (Pre-WroTy)
Samples collected in wrong container (Pre-WroCo)
Incorrect fill level (Pre-InsV)
Unsuitable samples for transportation and storage problems:
Samples not received (Pre-NotRec)
Samples transported inappropriately (Pre-DamS)
Samples with excessive transportation time (Pre-ExcTim)
Sample haemolysed (Pre-HemI)
Samples clotted (Pre-Clot)
**Analytical phase**
Test covered by an EQA-PT control (Intra-EQA):
Unacceptable performances in EQA-PT schemes (Intra-Unac)
Test with inappropriate IQC performances (Intra-Var)
**Postanalytical phase**
Critical values notification:
Critical values notified successfully (Post-SucCV)
Critical values notified within a consensually agreed time (Post-InpCv and Post-OutCV)
Inappropriate turnaround times (Post-OutTime)

### Statistical analysis

The results of the QIs were stated as in percentage (%) and sigma value. Sigma levels were calculated using Six Sigma calculators (*20*). The results of defect percentages and sigma values were presented as three levels of performance quality: 25^th^, 50^th^, and 75th percentile. The 25^th^ percentile exhibits the best performance (high); the 50^th^ percentile exhibits the common performance, and the 75^th^ percentile exhibits the worst performance (*13*). In our study, 6 months before the pandemic were considered as a group and 6 months after the pandemic onset (during a pandemic) as a separate group. To compare differences among groups Student’s t-test or Mann Whitney U test were applied. All the statistical calculations were conducted using the Statistical Package for Social Sciences (SPSS) software program (v.22; IBM, Armonk, NY) and a P<0.05 was established statistically significant for all analyses.

## Results

### QIs related to preanalytical phase

**Figure 1:**
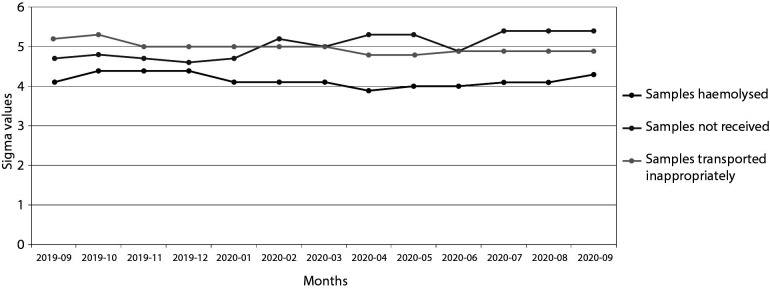
Sigma trend of quality indicators related to pre-analytical phase during the study period Sigma values of “Samples haemolysed”, “Samples not received”, and “Samples transported inappropriately” from six months before pandemic (March 2020 is recognized as beginning of pandemic) to six months after pandemic onset.

While an average of 8 tests were requested from 1 sample in the pre-pandemic period, this value was 13 during the pandemic. Defect percentages and sigma values related to pre-analytical phase were presented in Table 2 and Figure 1. The median defect percentages of these QIs range from 0.005% (misidentified requests) to 1.165% (anticoagulant samples clotted) for the pre-pandemic period and 0.002% (misidentified requests) to 1.198% (anticoagulant samples clotted) during pandemic. Meanwhile, median sigma values of the eleven QIs were all above 4.0σ except anticoagulant samples clotted both for the pre-pandemic period and during the pandemic group (3.8σ, and 3.9σ, respectively). While defect percentages of 6 of the 11 QIs (misidentified samples, inappropriate test requests, incorrect sample type, incorrect fill level, samples transported inappropriately, sample haemolysed) were found to have increased and sigma values were decreased during pandemic compared to the pre-pandemic. Among the 11 preanalytical QIs, only 2 defect percentages of them (misidentified requests and samples not received) were obtained to have decreased and sigma values were increased during pandemic compared to the pre-pandemic. Furthermore, defect percentages and sigma values of the 3 QIs (samples collected in the wrong container, samples with excessive transportation time, and anticoagulant samples clotted) were almost the same before and during the pandemic. In addition to these, when the pre- and during pandemic periods were compared, the defect percentage of the samples not received was significantly higher and the sigma value of the samples not received was significantly lower in pre-pandemic group than during the pandemic group (P = 0.006, P = 0.003, respectively). The defect percentage of the samples transported inappropriately was lower and the sigma value of the samples transported inappropriately was higher in pre-pandemic period than during the pandemic period (P = 0.004, P = 0.046, respectively). Also, the defect percentage of the haemolysed samples were significantly higher and the sigma value of the haemolysed samples were significantly lower during the pandemic period than the pre-pandemic period (P = 0.048, P = 0.044, respectively).

**Table 2:** Quality indicators reflecting preanalytical phase from pre- and during pandemic periods

			**Defect percentages, %**			**Sigma values**			**P**
**Code**	**Quality indicators**	**Period**	**25th**	**50th**	**75th**	**25th**	**50th**	**75th**
**Misidentification errors**									
**Pre-MisR**	Percentage of number of misidentified requests/total number of requests	Pre-pandemic	0.002	0.005	0.008	5.3	5.5	5.7	0.423*
		During pandemic	0.002	0.002	0.005	5.3	5.6	5.7	0.418^†^
**Pre-MisS**	Percentage of number of misidentified samples/total number of samples	Pre-pandemic	0.008	0.010	0.015	5.2	5.3	5.3	0.200*
		During pandemic	0.011	0.017	0.020	5.1	5.1	5.2	0.186^†^
**Inappropriate test requests**									
**Pre-OffDE**	Percentage of number of inappropriate requests/total number of requests	Pre-pandemic	0.044	0.051	0.059	4.8	4.9	5.0	0.150*
		During pandemic	0.046	0.087	0.140	4.6	4.7	4.8	0.061^†^
**Incorrect sample type**									
**Pre-WroTy**	Percentage of number of samples of wrong or inappropriate type/total number of samples	Pre-pandemic	0.312	0.421	0.593	4.1	4.2	4.3	0.521*
		During pandemic	0.267	0.82	1.445	3.7	4.0	4.3	0.287^†^
**Pre-WroCo**	Percentage of number of samples collected in wrong container/total number of samples	Pre-pandemic	0.076	0.091	0.096	4.7	4.7	4.7	0.199*
		During pandemic	0.064	0.076	0.104	4.7	4.8	4.80	0.207^†^
**Incorrect fill level**									
**Pre-InsV**	Percentage of number of samples with insufficient sample volume/total number of samples	Pre-pandemic	0.262	0.288	0.924	3.9	4.3	4.3	0.261*
		During pandemic	0.566	0.719	0.927	3.9	4.0	4.1	0.254^†^
**Unsuitable samples for transportation and storage problems**									
**Pre-NotRec**	Percentage of number of samples not received/total number of samples	Pre-pandemic	0.060	0.072	0.092	4.7	4.7	4.9	0.006*
		During pandemic	0.005	0.007	0.018	5.2	5.4	5.4	0.003^†^
**Pre-DamS**	Percentage of number of samples transported inappropriately/total number of samples	Pre-pandemic	0.015	0.027	0.032	5.0	5.0	5.3	0.004*
		During pandemic	0.042	0.045	0.054	4.8	4.9	4.9	0.046^†^
**Pre-ExcTim**	Percentage of number of samples with excessive transportation time/total number of samples	Pre-pandemic	0.007	0.008	0.013	5.2	5.3	5.4	0.810*
		During pandemic	0.006	0.007	0.019	5.2	5.4	5.4	0.935^†^
**Sample haemolysed**									
**Pre-HemI**	Percentage of number of haemolysed samples (clinical chemistry)/total number of samples (clinical chemistry)	Pre-pandemic	0.211	0.364	0.586	4.1	4.3	4.4	0.048*
		During pandemic	0.461	0.628	0.764	4.0	4.1	4.2	0.044^†^
**Samples clotted**									
**Pre-Clot**	Percentage of number of samples clotted /total number of samples with an anticoagulant	Pre-pandemic	1.014	1.165	1.179	3.8	3.8	3.9	0.630*
		During pandemic	0.820	1.198	1.373	3.8	3.9	4.0	0.727^†^
P < 0.05 statistically significance. *statistically significant difference of defect percentages between study periods. ^†^statistically significant difference of sigma values between study periods.									

### QIs related to analytical phase

The QIs related to the analytical phase listed in Table 3. Although the defect percentage of a number of unacceptable performances in External Quality Assessment or Proficiency Testing (EQA-PT) schemes during the pandemic period was higher and the sigma value of the unacceptable performances in EQA-PT schemes were lower compared to the pre-pandemic period, the differences between them were not significant (P = 0.317, P = 0.936, respectively) (Table 3). As with unacceptable performances in EQA-PT schemes, the defect percentage of tests with inappropriate internal quality control (IQC) performances was higher during the pandemic period compared to the pre-pandemic period, while the sigma value was lower. However, when the two periods were compared, no significant difference was found for this quality indicator (P = 0.699, P = 0.081, respectively).

**Table 3:** Quality indicators reflecting analytical phase from pre- and during pandemic periods

			**Defect percentages, %**			**Sigma value**			**P**
**Code**	**Quality indicators**	**Period**	**25th**	**50th**	**75th**	**25th**	**50th**	**75th**
**Test covered by an EQA-PT control**									
**Intra-EQA**	Percentage of number of tests with EQA-PT control/total number of tests available in an EQA-PT provider	Pre-pandemic	100	100	100	6.0	6.0	6.0	0.999*
		During pandemic	100	100	100	6.0	6.0	6.0	0.999^†^
**Unacceptable performances in EQA-PT schemes**									
**Intra-Unac**	Percentage of number of unacceptable performances in EQA-PT schemes/total number of performances in EQA schemes	Pre-pandemic	0.712	0.832	0.913	3.9	3.9	4.0	0.317*
		During pandemic	1.007	1.118	1.294	3.8	3.8	3.8	0.936^†^
**Test with inappropriate IQC performances**									
**Intra-Var**	Percentage of number of tests with CV% higher than selected target/total number of tests with CV% known	Pre-pandemic	2.78	4.25	5.91	3.1	3.3	3.4	0.699*
		During pandemic	3.09	6.38	6.94	3.0	3.2	3.3	0.081^†^
P < 0.05 statistically significance. *statistically significant difference of defect percentages between study periods. ^†^statistically significant difference of sigma values between study periods. EQA – External Quality Assessment. EQA-PT – External Quality Assessment or Proficiency Testing. IQC – internal quality control. CV – coefficient of variation.									

### QIs related to post-analytical phase

The selected QIs related to the post-analytical phase are shown in Table 1. While the critical value rate was 0.0996% before the pandemic, it was 0.1014% during the pandemic. Defect percentages and sigma values related to the post-analytical phase were presented in Table 4. There was no difference in the number of critical values successfully reported between the pre-pandemic and during pandemic periods (P = 1.000). Although the defect percentage and the sigma value of the critical values notified within a consensually agreed time decreased during pandemic compared to the pre-pandemic period, the difference between them was not significant (P = 0.631, P = 0.476, respectively). The defect percentage of the reports delivered outside the specified time was lower and the sigma value of the reports delivered outside the specified time was higher during the pandemic than the pre-pandemic period (P = 0.004, P = 0.030, respectively).

**Table 4:** Quality indicators reflecting postanalytical phase from pre- and during pandemic periods

			**Defect percentages, %**			**Sigma value**			**P**
**Code**	**Quality indicators**	**Period**	**25th**	**50th**	**75th**	**25th**	**50th**	**75th**
**Critical values notification**									
**Post-SucCV**	Percentage of number of critical values notified successfully/total number of critical values need to communicate	Pre-pandemic	100	100	100	6.0	6.0	6.0	0.999*
		During pandemic	100	100	100	6.0	6.0	6.0	0.999^†^
**Post-InpCV** **and** **Post_Out CV**	Percentage of number of critical values notified within a consensually agreed time/total number of critical values need to communicate	Pre-pandemic	96.34	98.75	99.02	3.7	3.8	3.8	0.631*
		During pandemic	95.89	97.72	98.17	3.6	3.6	3.7	0.476^†^
**Inappropriate turnaround times**									
**Post-Out Time**	Percentage of number of reports delivered outside the specified time/total number of reports	Pre-pandemic	7.93	8.42	8.96	2.9	3.0	3.0	0.004*
		During pandemic	5.57	6.81	7.07	3.0	3.1	3.2	0.030^†^
P < 0.05 statistically significance. *statistically significant difference of defect percentages between study periods. ^†^statistically significant difference of sigma values between study periods. CV – coefficient of variation.									

## Discussion

In this study, we examined the response of the total testing process in laboratory medicine to the COVID-19 pandemic depending on possible laboratory errors related to changes in laboratory process during the pandemic period. We sought an answer to the question of how the COVID-19 outbreak affected the TTP by comparing quality indicators in the pre-pandemic period with during the pandemic. Certain effects of the COVID-19 pandemic on the TTP were noted. In all TTP phases, some quality indicators improved while others regressed during the pandemic.

The COVID-19 pandemic continues to spread the whole world and poses a threat to communities and health systems (*21*). Due to the increasing number of SARS-CoV-2 cases, the high workload of the laboratory staff, and the enhanced pressure, the laboratory test process is sensitive to errors (*22*). Errors in laboratory testing can lead to critical influence on patient care. To monitor errors in TTP, QIs are used in clinical laboratories (*23*). When faced with natural disasters such as epidemics, tsunamis, earthquakes, we do not have much information about what awaits us in clinical laboratory medicine. To the best of our knowledge, no prior report related to the effect of the COVID-19 pandemic on TTP has been published.

Certain quality indicators improved while others regressed during the pandemic period. Since most of the patients who come after the pandemic are SARS-CoV-2 infected, the number of misidentified requests may have decreased due to the request made in the test panel determined specifically for them. There were deteriorations in the performances of misidentified samples and inappropriate test requests after the pandemic. This may have resulted from sampling with minimal contact in a minimum time due to increased workload and contamination risk. The rise of errors in process regarded with wrong or inappropriate sample type can be due to an increase in the requests of the coagulation tests during the pandemic period. Although the total number of tests before and during the pandemic is almost the same, the number of tests run from one sample has increased considerably, causing enhanced rejection of insufficient sample volume. The performance of samples not received was significantly improved from the pre-pandemic period to during the pandemic. This is most likely due to the low number of samples during the pandemic. There was a significantly worsening in the performance of samples transported inappropriately. This worsening may be the result of the insufficient number of staff during the pandemic. During the pandemic, healthcare staff may also be temporarily moved from one unit to another in the hospital. Also, there was a large number of blood collection staff who had a confirmed SARS-CoV-2 infection and had to stay under treatment and take a break from their duties. The use of syringes increased when the holder became insufficient in blood collection because of the disruptions in the supply chain during the pandemic and all these may have an impact on the enhanced rate of the samples haemolysed.

The error rates of the intra-analytical phase vary from before pandemic to during pandemic. Although the number of unacceptable performances in EQA-PT schemes increased slightly during the pandemic, this increase was not significant. The defect percentage of the tests with inappropriate IQC performances was found to be higher during the pandemic. The temporary movement of laboratory staff from one laboratory to another (*e.g.*, from biochemistry to a virology laboratory), flexible working application in the laboratory, need for additional equipment up to the excessive increase in some parameters, additional personnel need, and device user changes may have led to an increase in manual laboratory errors. These results indicate that there is a need for improvement in the analytical process.

Decreased errors in the post-analytical process promote and improve the patient’s safety. Since our hospital is a pandemic hospital, the slight decrease in the number of critical values notified within a consequently agreed time during the pandemic may be due to the increase in critical value rates with the effect of COVID-19 patients. The decrease in the number of samples reaching the laboratory during the pandemic period and the excessive effort of the laboratory staff during the pandemic process may have developed the performance of turnaround times. In order to contribute to patient care, the quality in the post-analytical phase should be improved and monitored.

Lippi *et al.* have been identified the potential preanalytical and analytical susceptibilities in the laboratory diagnosis of COVID-19 to reduce the risk of diagnostic errors and to improve diagnostic accuracy (*22*). The authors mentioned that the susceptibility of laboratory medicine is greatly increased when staff are forced to work in high-productivity environments to face high workloads and are forced to work under severe pressure with the increasing number of positive cases of SARS-CoV-2 requiring comprehensive health support (*22*). Similarly, we identified laboratory errors caused by high workloads and severe pressure during the pandemic process. Taylor *et al.* have evaluated the effects of an earthquake on turnaround times (TATs) at a laboratory (*24*). They have found increased registration and transport time of the potassium test, but no significant impact was observed regarding their analysis time. On the contrary, in our study, due to the decreased sample number, a decrease was observed in the turnaround time during the pandemic compared to the period before the pandemic. Lyon *et al.* applied simulation models to estimate test capacity during the COVID-19 pandemic (*25*). They simulated six workload conditions. In this study, the 90% percentile TATs are expected to remain stable until the analytical system’s maximum throughput is overcome (*25*). One disadvantage of this simulation can be that parameters such as the number of laboratory staff and adaptation to excessive workload cannot be evaluated. However, in our study, we can clearly see the effects of all parameters such as staff and workload during the pandemic period.

However, there were certain limitations in this study. Firstly, more than 17 QIs could be used to assess the impact of the COVID-19 outbreak on the TTP. Though, the choice of these 17 QIs was widely based upon the power of a comprehensive evaluation of the most error-inclined testing process and their potential impact on patient safety. Secondly, the performance level of QIs of other clinical laboratories of pandemic hospitals could be included in the investigation, and data from all pandemic hospitals could be compared.

In conclusion, in all TTP phases, some quality indicators improved while others regressed during the pandemic period. The maximum variation in the performance of QIs was observed in the pre-analytical phase. The most notable alteration seen in the pre-analytical phase was unsuitable samples for transportation and storage problems. The most remarkable effect of the COVID-19 outbreak in the post-analytical phase was the reduction of turnaround times.

This research highlights the areas that need development when faced with natural disasters such as viral pandemics. While the availability of test reagents, laboratory equipment, and qualified laboratory staff is important, stress and increased workload during the crisis also make laboratory medicine susceptible to laboratory errors. So, the take-home message here is, detecting laboratory errors, correcting them, and taking timely action is very important in achieving control of the crisis. Monitoring laboratory errors with QIs is likely to take measures to manage future crises. Laboratories should have plans of action for several predicaments. Education for the pandemic procedures should be standard procedure in laboratory medicine and laboratory staff should be aware of necessary procedures.
